# Stimuli-Responsive Polymeric Nanosystem for Colon Specific Drug Delivery

**DOI:** 10.15171/apb.2020.001

**Published:** 2019-12-11

**Authors:** Sharon Kunnath Joseph, Mangalath Sabitha, Sreeja Chandrasekharan Nair

**Affiliations:** Amrita School of Pharmacy, Amrita Vishwa Vidyapeetham, AIMS Health Sciences Campus, Kochi-682041, India.

**Keywords:** Nanopolymers, Stimuli responsive polymers, Controlled release, Nano platform, Smart polymers, Colon specific drug delivery

## Abstract

An ideal colon specific drug delivery system needs to perform multiple functions like greater bio availability, less toxicity and higher therapeutic efficacy, all of which require high degree of smartness. This article focuses on the overview of the stimuli-responsive polymers and various nanodrug delivery systems which have found applications in colon specific delivery of drugs as this system provide a link between therapeutic need and drug delivery. These polymers exhibit a non-linear response to a small stimulus leading to a macroscopic alteration in their structure/properties. Stimuli responsive polymers display a significant physio chemical change in response to small changes in their environment (temperature, pH, light etc.). Colonic drug delivery has gained increased importance in treating diseases like Crohn’s disease, ulcerative colitis, colon cancer etc. The expansion in the development of polymers based system with greater flexibility, versatility and unexplored potential enables new opportunities for them in uplifting bio medicine. Applying the concepts of smartness in the context of clinically relevant therapeutic and diagnostic systems, it can prelude in a new era of ‘smart’ therapeutics that can improve the health care fields. In particular, due to its high sensitivity to the stimuli, this system has been identified as a sensible platform for releasing drug at suitable site and at appropriate time.

## Introduction

Targeted drug delivery system (TDDS) is a special type of drug delivery system that enhances the therapeutic effects by specifically targeting the diseased tissue or organ to deliver the medicament avoiding the hepatic first pass metabolism and enhancing therapeutic index. This system can provide an alteration in its own property in response to environmental changes.^1^ Before the advancement in science field, conventional drug delivery systems (CDDSs) posed a great challenge to researchers as they often cause some sort of systemic side effects because of their nonspecific bio distribution and uncontrollable drug release characteristics.^2^ In order to address these problems, it was essential to enhance the efficacy, bio compatibility and reduce the side effects; also the active drug molecule should specifically accumulate in targeted or diseased area for a longer period with high controllability.^3^ Polymeric systems, especially smart system with intelligent technologies have been developed for colon targeting that holds a great potential in nanomedicine. In polymeric nanosystems, drugs are released in sustained and controlled manner with the ability to integrate both hydrophilic and hydrophobic drugs. But polymeric nanosystems have its own limitations like inability for their scale-up and lack of proper toxic valuation in the literature. Nano-dimensional systems have shown the capability to enter cells and its components but the delivery of nano particles to some particular tissues and organs remains a problem due to various biological barriers present. Example, crossing the lung tissues or pleural walls or the gut.^4^ Further modifications in shape, solubility, mobility, localization, swelling characteristics, interactions with molecules etc can enable them to conquer the biological and physical barriers (the barriers include the mucus barrier at the epithelial surface, the bestirred water layer present between the mucus layer and epithelial cells, the lipid bilayers of the individual colonocytes and the occluding junctional complex between these cells etc) and thus displays environment driven response and functions. These responses can be exploited for therapeutic and diagnostic applications.^5^

Diseases related to colon like ulcerative colitis pose a challenge to many across the world where the only alternative available for symptomatic relief was anti-inflammatory agents and some corrected with surgical procedures. The glitch arose due to inappropriate drug delivery to target site and improper concentration of the drug at the target site, so in order to address these, the new targeted polymeric drug delivery system has been discovered to get an efficient therapeutic goal.^6^ This drug delivery system pursue to concentrate the drug in the diseased tissues or the tissues requiring the pharmacological effect, either passively or actively, after its administration in smaller doses. Thus, it reduces the freely circulating drugs at non target sites or minimizes the relative concentration of the medication in the remaining tissues which ultimately paves a way for decreased side effects, toxicity and undesirable interactions at other sites.^7^ Table 1 shows the comparison between conventional and targeted polymeric drug delivery system.^8^ This system respond to various stimuli that can be classified under two categories:

**Table 1:** Comparison between conventional and targeted polymeric drug delivery system

**Conventional drug delivery system**	**Targeted polymeric drug delivery system**
Affect healthy tissues/organs	Do not affect healthy tissues / organs
Bioavailability is low	High bioavailability and biocompatibility
Non specific	Specific
Lower efficacy and therapeutic effect	Higher efficacy and therapeutic effect
Toxicity level is higher	Toxicity level is lower
High dose required	Low dose required
Higher side effects	Lower side effects

Exogenous stimuli (electric pulse, temperature, ultrasound, magnetic field, etc) and Endogenous stimuli (hormone level, pH alterations, enzyme concentration, small biomolecules, glucose etc).^9^

Due to its specific action, smart drug delivery system has many applications in the treatment of cancer, cardiovascular diseases, diabetes, tumors and intestinal inflammation. It has also proved to be beneficial in colon specific smart drug delivery system.^10^

## Anatomy of colon

**Figure 1:**
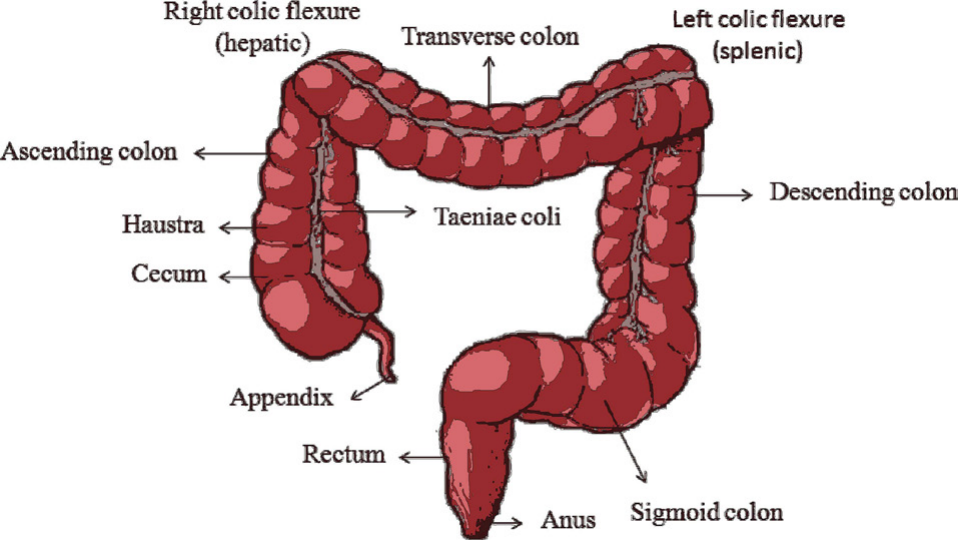
Anatomy of colon.

Alimentary canal comprises parts from mouth to anus. It measures about 5 m long in which the contribution of large intestine is 1.5 m (5 feet). Serosa, muscularis, submucosa, mucosa are the histological structures of the colon where the supra mesenteric artery supplies blood to the proximal colon.^11^ The large intestine which is a distal part (blood supplied by the inferior mesenteric artery) of the gastrointestinal tract bind around the frontier of the abdominal cavity from the lower right side of the body which is connected to the ileum of small intestine via ileocecal sphincter, across the top of the abdomen and then to the lower left side. Structurally, it is divided into 4 main regions namely cecum, colon, rectum and anal canal, shown in Figure 1.^12^ Cecum is a small pouch like structure of about 6 cm long which hangs inferior to ileocecal valve. Cecum opens to a long tube like structure called colon that is embryonically developed partly from mid gut and hind gut. It is divided into 4 parts ascending colon, transverse colon, descending colon and sigmoid colon. Ascending colon on the right side of the body takes a 90 degree turn below the liver (hepatic flexure or right colic) and becomes transverse colon which extends from right to left and again takes a 90 degree turn below the spleen (splenic or left colic flexure) becoming the descending colon. The descending colon leads to the sigmoid colon which has an inverted V-shape. It is then followed by rectum which lies anterior to sacrum and coccyx and ends in anus.^13^ Large intestine is the reservoir of a number of bacteria which plays a beneficial role in our body to digest substances creating gas and making substances like vitamin K (helps in blood clotting).^14^

## Diseases related to colon

### Inflammatory bowel diseases

The two inflammatory bowel diseases (IBD) that lead to chronic inflammation and associated extra intestinal manifestations in the gastrointestinal (GI) tract are Crohn’s disease and ulcerative colitis. The major symptoms associated with IBD are abdominal cramps and pain, diarrhea, weight loss and bleeding from intestine. More than 600 000 Americans have some kind of IBD every year.^15^

#### Crohn’s disease

Crohn’s disease is a disease characterized by inflammation or swelling in any part of GIT mostly rectum. The inflammation can become so intense that it can penetrate the lining of the affected part. The result of the disease could be pain and diarrhea. Scar tissues are produced when the inflammation is chronic. The factors affecting this disease are genetic, immunologic, infective agents. Cyclosporin is the drug which is used orally to treat the disease.^16^

#### Ulcerative colitis

In this disease inner linings of large intestine, usually the lower section, colon and rectum is affected which produces inflammation and ulcers or sores.^17^ This disease usually begins in the rectal area and extends through the entire large intestine. Repeated inflammation leads to thickening of the wall of the intestine and rectum with scar tissue. The symptoms of this disease are bleeding, abdominal discomfort and diarrhea.^18^

### Colorectal cancer

Colorectal cancer is the cancer that starts either in the colon or the rectum. Appearance of blood in stool, diarrhea, constipation, abdominal pain, cramps, blotting, weight loss, feeling of fatigue are the symptoms of the disease.^19^

The smart drug delivery system has proved clinical benefits in the treatment of above mentioned colon diseases.

## Factors affecting colon drug delivery

They are many factors affecting colon drug delivery which are categorized into two: physiological and pharmaceutical factors.

### Physiological factors

#### Transit time in intestine and colon

According to the research conducted by Mayo Clinic researchers in 21 healthy people, it showed that the transit time averaged 53 hours in total. While comparing with other parts of GI tract, passage of any material through colon is very slow and takes about 40 hours.^20^ Studies by Rana et al show that the transit time of UC or IBD patients change evidently.^21^ For entering the colon in appropriate form, the drug delivery system must go beyond all the barriers in stomach and small intestine. The dosage form takes about 3-4 hours to reach ileocecal junction.^22^ Presence of certain enzymes in small intestine can impose changes in the dosage form. The release of medicament from dosage form is controlled by factors like microbial flora in the colon, habitation enhancement with longer passage time.^23^

#### Gastric emptying

Gastric emptying and passage of bowel plays an important role in governing drug delivery to the colon by oral administration. The matter of issue when the dosage form reach the stomach is the duration in which it will remain in the stomach before reaching the duodenum.^24^ The gastric emptying process varies according to the phases of stomach when the drug is administered. Once the drug reaches the colon, particle size plays a key role in judging the transit time of dosage form. Usually smaller particles can easily pass through the colon than larger ones. It usually completes in 5-10 minutes up to 2 hours.^25^ A colonic drug delivery system is efficient if it remains only for some time in stomach. Thus, an efficient delivery system can release drug at a far place from the colon. A person affected with diarrhea have little transit time while a person affected with constipation have larger transit time.^26^

#### Stomach and intestinal pH

The presence of enteric coatings on the drugs make the pH of the GIT an important factor. In a healthy adult the pH in stomach ranges from 1.5 to 3.5 which is highly acidic and in duodenum the pH increases to 6. Gradually pH reaches 7.4 in the small intestine and then drops to a pH range 5.5-7 in the colon but this can differ depending on the individual variations, presence of food, condition of person whether healthy or diseased etc.^27^ Colon drug delivery is formulated mainly based on the pH and this pH gradient triggers the drug release. A drug is usually enclosed by a polymer coating to aim the drug at specified location, to protect the acid labile drugs from gastric fluid and to prevent GI disturbances due to irritation from the drug.^28^ Some polymers used for enteric coating are hydroxypropyl methyl cellulose phthalate, polyvinyl acetate phthalate, cellulose acetate phthalate and acrylate polymers which disintegrate only in the intestinal pH thus enhancing drug bio availability specifically at colon.^29^

#### Colonic microflora and enzyme

The GIT is often referred to as the store house of a large number of microorganisms that are capable of producing several enzymes which can accelerate metabolism process. These micro flora of the colon holds a variety of applications in health and for treating GI disorders like IBD.^30^ Peristaltic movement and the contents in GIT control the growth of this micro flora. The increased concentration of micro flora becomes prominent in the terminal ileum and lead to high levels in the colon. The microbial bacteria have the potential to catalyse an enormous number of metabolic activities in the body.^31^ The enzymes produced by microbial flora mainly glycosidase and azoreductase posse the ability to release the drug in the colon. Sulphasalazine, a sulphonamide used for intestinal infection, is a prodrug that is converted to m-amino salicylic acid (having an anti-inflammatory effect on colon; prevents systemic absorption and hence increases the duration of action) and sulphapyridine (antibacterial effect) by azoreductase. Gut micro flora actively hydrolyzes a large number of polysaccharides which leads to the opportunity of making drug carriers out of naturally occurring bio polymers.^32^

#### Gastrointestinal disease condition

The existence of different gastrointestinal diseases like IBD, constipation, diarrhea alter the drug delivery system in colon.^33^

IBD can reduce the surface area and decrease the diffusion rate of drugs due to the thickening of mucosa and submucosa thus leading to malabsorption of lipophilic drugs. While diarrhea reduces the retention time and hence decreases the drug absorption and release from dosage form.^34^

#### Pharmaceutical factors

#### Drug candidates

An increase in absorption of weakly absorbed agents like peptides occurs because colon offers a high time for these agents to stay in it. Drug molecules used to cure GIT disorders can also be used for colon targeted drug delivery.^35^

#### Drug carriers

The factors like drug nature, its indication are used a tool for selection of CDDS carriers. Apart from this, other factors involved are functional group of drug molecule, chemical nature, its stability factor, constant factors like partition coefficient etc.^36^

## Different Approaches for Colon Drug Delivery

### Prodrug

Prodrug is a part of parent drug that possess no activity but has the ability to get converted to its active form by undergoing enzyme transformation and get released at the target site.^37^ This is used when a drug have undesirable physicochemical property. In this process, drug and its carrier are joined by covalent linkage and when the drug is administered via oral route, it remains confined to stomach and intestine. Pectin-metronidazole is an example of prodrug which is unaffected by the acidic pH of stomach and enhance the delivery of drug directly to colon ensuring higher bio availability.^38^

### Azo bond conjugate

The azo compounds can act as drug carriers for colon related drug targeting and are used in hydro gel form.^39^ For the treatment of IBDs, Sulfasalazine is used which contain an azo bond between 5-aminosalicylic acid (5-ASA) and sulfapyridine (SP). The enzyme azoreductase present in the intestine cleaves the azo bond and thus releases the drug 5-ASA and carrier SP.^40^ With the advancement in scientific arena steroid glycosides and the glycosidase activity have proved an essential role in colon TDDS. The principle lies on conjugation of different drug to different sugar moieties and ultimately forms glycosides.^41^ There are two parts for the compound: aglycone which forms drug part and glycone which form sugar part. The glycosidase enzyme act on glycoside to release drug part and the added advantage of the enzyme is that it is easily available to the substrate since it is present at the brush border site.^42^ Some examples of glycosidase enzymes produced by intestinal microbial flora are beta-D-xylopyranosidase, beta-D-glucosidase, alpha-L-arabinofuranosidase, beta-D-galactosidase etc.^43^

### Glucuronide conjugate

The enzyme b-glucuronidase which deglucuronidate many drugs is secreted by bacteria present in the lower GIT.^44^ The process of deglucuronidation helps in the release of drug (active) and helps in its reabsorption.^45^

### Cyclodextrin conjugate

This type of oligosaccharide is cyclic and has an inner (lipophilic in nature) and outer part (hydrophilic in nature).^46^ They are composed of 6-8 glucose units which are linked through 1,4-glycosidic bond. The bond ensures high solubility, bioavailability and stability of the drug which has been utilized in colon TDDS.^47^

### Dextran conjugate

These are polysaccharides composed of monosaccharide joined together by glycoside linkage. The enzyme dextranase hydrolyses the linkage producing shorter prodrug oligomer which is further cleaved by colonic esterase to release free form of drug to colon.^48^ The activity of dextranase is evident in colon but its activity is almost nil in upper part of GIT. Drug possessing carboxyl group can be targeted for colon drug delivery using dextran prodrug. Prodrug of nalidixic, dextran - nalidixic acid ester (dextran-NA) with a varied degree of substitution was synthesized for colon specific action.^49^

### Polymeric prodrugs

Researchers are trying to expand the role of polymeric prodrugs like synthetic as well as natural polymers to carry out the drug delivery to colon. A few polymeric prodrugs commonly used are: polyethylene glycol, divinyl ether malic anhydride/acid polymer, polyethylenimine etc.^50^ An azo linkage is present between the drug and the polymer used. Studies were done on this aspect and it showed that a buffered solution or solution containing pancreatin is used to find out the extent to which destruction of drug occurs in the intestine. These studies also demonstrated that several factors like type of monomer, type of bond between the polymer and drug; and swelling factor are responsible for its proper action at the target site.^51^

### Antibody targeted smart polymer system

Researchers are trying to adopt antibody mediated polymeric system for aiming efficient colonic drug delivery. Mane and Muro have investigated about this method and explained their experiment using anti-ICAM- antibodies which were surrounded over the nanoparticles.^52^ On the oral administration of this antibody coated nanoparticle, it was found that after the exposure to enzymes present in GI tract, these antibodies were destructed while the nanoparticles got lodged in the duodenum. These scientists used techniques like transmission electron microscopy and energy dispersive X–ray in their experiments.^53^

## Rationale of different stimuli responsive polymers towards colon

### Microbially triggered system

As the name suggests, these are the systems that are triggered by microbial flora. The enzymes produced by the microbial flora present in the intestine disintegrate the biodegradable polymers after the coated oral formulation reaches the intestine and thus drug is released to the specified location.^54^ The drug coated with this polymer remain unaffected as it passes through stomach and small intestine since there are a very few microbes present in these regions. A study carried out by Hita et al revealed that azo aromatase polymers coated metronidazole capsules were active in treating colon related diseases and the enzymes present in the intestine degrade the polymers thus releasing the drug metronidazole locally in the colon.^55^

Bacteroides, bifidobacteria, eubacteria, enterococci,*Ruminococcus,* Clostridia etc are the different microbial population in the colon. These microflora perform fermentation by producing enzymes like galactosidase, xylosidase, arabinosidase, glucuronidase dehydroxylase, nitroreductase etc.^56^

### Osmotic controlled drug delivery system

In order to achieve systemic absorption of the drug for curing diseases related to colon, osmotic [controlled] release oral [delivery] system (OROS-CT) proves to be a viable method. The OROS-CT system comprises a single osmotic unit or may contain as many as 5-6 push-pull units, which are encapsulated within a hard gelatin capsule.^57^ The push pull unit consist of osmotic push layer and drug layer which form a bilayer. Both the layers are enclosed by a semi permeable membrane which contains an orifice near the drug layer. The gelatin capsule containing the push-pull units are dissolved after the administration of OROS-CT orally.^58^ The presence of drug-impermeable enteric coating in push-pull unit will not allow water to get absorbed in acidic nature of stomach and thus delivery of drug gets prevented. The presence of a higher pH (greater than 7) in small intestine causes the coating to dissolve and allows the water to get into the unit.^59^ Entry of water leads to the swelling of osmotic push compartment and then creates a flow able gel in the compartment. The drug gel is pushed out of the orifice when the osmotic push compartment swells up.^60^ It forces out at a rate accurately regulated by the rate of water via semi permeable membrane. For the treatment of ulcerative colitis, the unit is proposed in such a way so as to avoid the drug delivery in small intestine with a few hours of post gastric delay.^61^ When OROSCT units reaches the colon, it initiates the drug release and maintains a constant rate for drug release for up to almost 1 day in the colon or can target drug for a duration of few hours.^62^ With the advancement of technology new system has arrived which promises to be a better platform for drugs delivery to the colon. Many assessment methods are invented to test the efficiency and sensibility of CDDS.^63^

### pH responsive system

The pH of our empty stomach is between 1 and 2 but after the ingestion of food pH starts to rise. The pH of proximal small intestine is 6.5 and in the cecum it is about 6.4 while the transverse colon has a pH of 6.6 and descending colon has a pH of about 7.0.^64^ Table 2 shows the commonly used pH dependent polymers for various drugs in colon drug delivery.^65^ Eudragit L 100 and Eudragit S 100 are solid white powders with faint characteristic odor and they are chemically anionic copolymers based on methacrylic acid and methyl methacrylate while Eudragit RS 100 is a solid colorless, clear to cloudy granules with faint amine like odor and chemically a copolymer of ethyl acrylate, methyl methacrylate and a low content of methacrylic acid ester with quaternary ammonium groups. Eudragit polymer coating finds application in gene and vaccine delivery, ophthalmic, buccal and sublingual, transdermal, vaginal, intestinal, gastrointestinal and colon drug delivery.^66^ In colon drug delivery, they played a role in treating ulcerative colitis, Crohn’s disease and IBD and the drug used for IBD was tegaserod maleate. These pH responsive gels depending on the pH change gets protonated or deprotonated. The swelling behavior of the gel is modified by the protonation and deprotonation which alters the attractive forces between each polymeric chain in the gel.^67^ Poly(acrylic acid), poly(methacrylic acid) and alginate gel swells the maximum in alkaline pH whereas gels of chitosan, poly(L-lysine), poly (vinyl pyridine), poly(2-diisopropylaminoethyl methacrylate) etc swells in the acidic pH. Repulsive forces are experienced in the neighbouring polymer chains when they are in their charged states. In order to equalize the repulsion, the counter ions enter into the gel matrix from the medium along with water molecules which causes the gel to swell.^68^ This system has exploited well for drug delivery to colon as well to complete GIT. When considering an example, the pH responsive nanogel system contains N,N’ dimethyl acrylamide, t-butyl acrylamide and a copolymer of acrylic acid which is cross linked with N,N’-diaminocaproyldiaminoazobenzene and loaded with the desired drug.^69^ On administering it orally, it passes through different pH in the GIT (stomach 1-3; small intestine 4.8-8.2) which results in mild swelling and protonation and deprotonation respectively. As the nanoparticle reaches the colon, it disintegrates in the presence of azoreductase enzyme secreted by the bacterial colony residing in the colon.^70^ Studies on mouse colon carcinoma models were demonstrated by Wu et al and Ziong et al using pH sensitive hydrogel loaded with metformin and 5-fluorouracil; and doxorubicin respectively were found effective for the treatment of colon cancer.^71,72^

**Table 2:** pH dependent polymers for different drugs in colon drug delivery

**Polymer**	**Drug**	**Threshold pH**
Eudragit L100 and S100	Flurbiprofen, Mesalazine	6-7
Eudragit L100	Ibuprofen	6
Eudragit L100 and S100	Diclofenac sodium and 5-ASA	6-7
Eudragit RS 100	Paracetamol, dicyclomine, 5-fluorouracil	<6

### Pressure controlled drug delivery system

Peristaltic movement is the wavelike contractions mainly in the digestive tracts which help in the pushing of ingested food towards the anus. This movement of the intestine creates an increase in the luminal pressure which led to the development of pressure controlled drug delivery system.^73^ It has a drug containing gelatin capsule along with a suppository base which contains an insoluble polymer inside. The insoluble polymer can be ethyl cellulose; whose thickness decide the disintegrating ability of the capsule.^74^ After the administration of the capsule, the base gets dissolved due to the body temperature and absorbs the water content from the intestinal material which increases the viscosity and this result in the elevation of pressure which forces the capsule to expel out the drug content in the colon.^75^

### Temperature sensitive drug delivery system

Temperature sensitive drug delivery system is one of the important class of smart drug delivery system that direct towards the targeted tissue as a result of external or internal stimuli. This system encourages the development in biomedical field as they offer least toxicity, better therapeutic efficacy and decreases the damage to healthy tissues. It also plays a major role in targeting the tumorous cells and thus potentiating their treatment.^76^ Tumor cells have a temperature slightly greater than the normal healthy tissues, this principle is applied in the treatment to enhance the drug permeability to the site of cancerous cells. The thermo sensitive drug delivery system has been applied to liposomes which causes the release of drugs at a temperature of 42°C and resulted in maximum drug concentration in the tumor site.^77^ This drug delivery system can be applied to hydrogels also, a preclinical study has been conducted in a group of human colon cancer induced mice which showed a decrease in tumor volume 3 weeks after the intratumoral injection of thermosensitive hydrogel loaded with paclitaxel. The drug acted only on the tumor cells and did not affect the normal healthy tissues. An intramuscular injection of Silibinin coated poly (organophosphazene) hydrogel demonstrated an evident decline in the proliferation of colonic cancer cells within 40 days *in vivo.* ^78^

### Smart drug delivery systems

**Figure 2:**
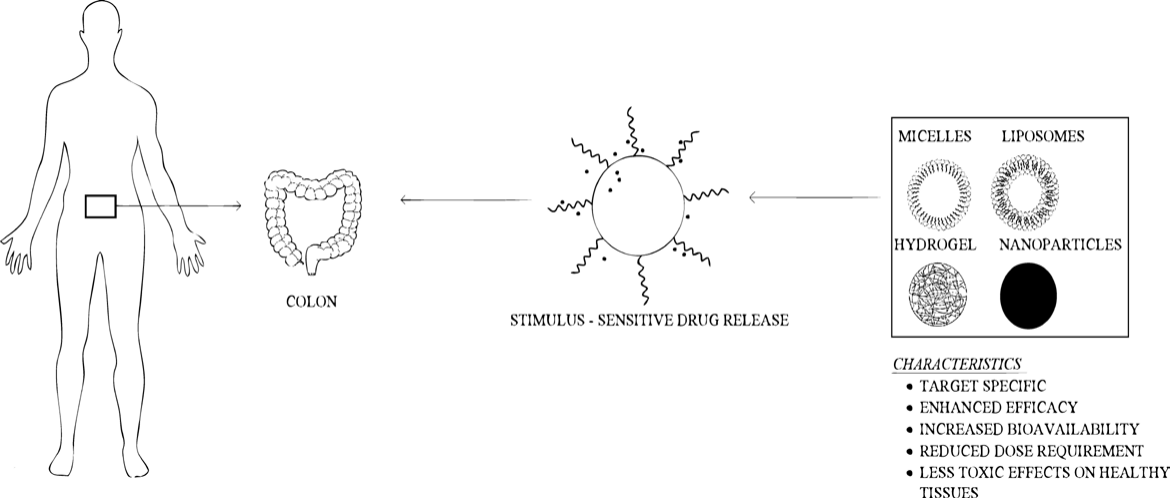
Diagrammatic illustration of smart drug colon drug delivery.

The application of smart polymers are utilised in nanoparticle drug delivery system. Table 3 shows different smart polymer based drug delivery systems especially for colon drug delivery^79^ and Figure 2 shows diagrammatic illustration of smart colon drug delivery.^80^ Two types of pH sensitive smart polymers are present which include anionic pH sensitive smart polymer and cationic pH sensitive smart polymer. The former includes poly(N,N-dimethylaminoethyl methacrylamide), polysulfonamide etc and the latter include poly(lysine) (PL), chitosan, poly(ethylenemine) etc.^81^

**Table 3:** Smart polymer-based drug delivery system for colon drug delivery

**Polymer**	**Drug**	**Type**	**Inventor**
Polyvinyl alcohol	Indomethacin	Hydrogel	Morimoto et al
Collagen -poly(HEMA)	5-FU	Hydrogel	Jayenthi et al
Chitosan –PEO	Amoxicillin and Metronidazole	Hydrogel	Patel et al
Hyaluronic acid	Danazol	Hydrogel	Normura et al
Chitin	Curcumin	Nanogel	Sabitha et al
Ethyl ethylene phosphate	Doxorubicin	Nanogel	Juan et al.
Chitosan graft poly(N-isopropylacrylamide	Oridonin	Nanogel	Duan et al.
Poly(ethylene glycol) methyl ether methacrylate(PEGMA)	Doxorubicin	Nanogel	Han Wen et al

#### Colon specific drug delivery using smart nano particles

Many studies have shown that drug delivery to colon using smart polymers have been effective in treating various IBD’s like Crohn’s disease, ulcerative colitis etc.^82^ For example, a hydrogel based targeting mechanism study on DSS mice (dextran sulfate sodium induced colitis) was carried out using poly-lactic acid nanoparticles loaded with tripeptide KPV (Lys-Pro-Val). It showed that, by using the nanoparticles, KPV was delivered at a concentration that is 12 000 fold lower than the KPV in free solution but provided similar therapeutic efficacy and reduced systemic side effects.^83^ An added advantage of drug delivery using nanoparticles is that their size results in the targeting drug to specific part thus enhancing bioavailability. A lower drug concentration can improve high therapeutic outcome which marks it as an outstanding system while conventional modes of treatment of these diseases using steroids like budesonide, beclomethasone (on prolonged use) led to systemic side effects.^84^ Usage of antibiotics (infliximab, adalimumab, golimumab etc), aminosalicylates, immunosuppressive agents were also a part of conventional mode of treatment. Oral drug delivery has increased the bioavailability of drugs to colon. Thus, conventional method do not guarantee to take the drug molecule to the diseased region and show its effect but a nanodrug delivery system shows best result in improving the target of drug to specified region ensuring high efficacy and less toxicity.^85^

#### Nanodrug delivery system based on size

**Figure 3:**
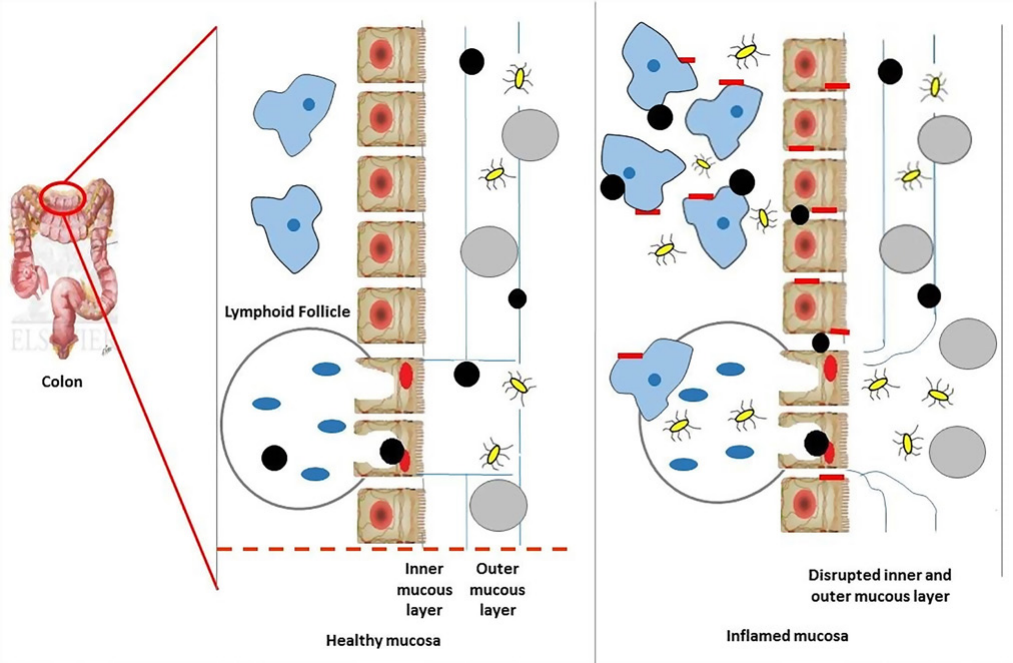
Characteristic changes in the binding of nanoparticles to healthy and inflamed mucosa in colon.

Minimizing the size of nanoparticles have shown a plenty of advantages to colon specific drug delivery. These include enhancing colonic residence time in affected regions, selective transport of drug molecules to diseased tissues by inducing an epithelial enhanced permeability, helping the uptake of nanoparticles by immunized cells which show larger counts in the damaged site.^86^ Nanodrug delivery system ensures rapid uptake of particles into inflamed tissues as shown in the Figure 3 and thus prevent rapid carrier elimination while conventional method focus only on ensuring regional drug deposition in GI tract.^87^ When nanoparticles reach GI tract, they undergo a process called cellular internalization by endocytosis into the epithelial cells in GI tract. Micro fold cells (M cells) present in the epithelial cells are responsible for the uptake of nanoparticles in IBD.^88^

A healthy mucosa contains a normal protective inner and outer mucous layer which is absent or comparatively less protective in inflamed mucosal layer. The M cells in both healthy and affected mucosal layer contain no mucosal gel layer as protective covering and are targeted by nanoparticles.^89^ In the affected mucosal layer, along with loss of mucosal gel layer, there is destruction of barrier of epithelium through the damage of enterocytes and there is increased transfer of microbial flora in intestine which leads to transport of immunized cells to the layer of mucosa. Thus, nanoparticles are taken up by enterocytes and macrophages and makes nanoparticles vulnerable to inflammatory receptor targets.^90^ The accumulation of nanoparticles was possible only because of its particle size and decreased diameter.^91^ A study was performed to support the above fact, a fluorescent polystyrene particle ranging from 0.1 to 10 μm in size was administered orally *in vivo* for 3 days and it was found that particle of range 0.1 μm had the highest binding affinity to affected tissue. Also, the rate of colitis or control deposition was increased by reducing particle size.^92^ The total amount fluorescence which is present in tissue is decreased by approximately 39% after mucus has been removed from inflamed tissue by repeated steps of washing. This means that large number of particles was bound to layer of mucosa which is insoluble than being uptaken by macrophage. Passive nanoparticle targeting enhances retentivity and permeability of drug but can lessen the selectivity of drug i.e., it is accessed to healthier tissue along with diseased tissues.^93^ For example, cetyltrimethyl ammonium bromide coated nanoparticle was bound to both inflamed and non-inflamed colonic tissue.^94^ Conversely, nanoparticles made with polyactide co-glycolide containing tacrolimus an immunosuppressant drug enhanced drug adherence to only inflamed tissue.^95^

#### Nano drug based on surface charge

Changing the surface charge of nanoparticles can affect the electrostatic interaction of nanoparticles with components in GI tract.^96^ The nanoparticles also have affinity towards charge modifying substances like bile acids, soluble mucins etc, during GI transit. Thus, apart from existing strategies, additional methods are needed to confine the drug delivery to diseased region.^97^

#### Nano drug delivery system based on positive charge – mucoadhesive

Nanoparticles coated with positive charge attract to mucosal surface in the inflamed region due to interaction of positively charged nanoparticle and negatively charged mucosal layer.^98^ Mucins present in colon are negatively charged because of presence of sulfates and scales acid in the carbohydrates.^99^ In Crohn’s disease, there is an increase in mucus production thus paving way of attachment of nanoparticles to thick mucus layer more strongly and leads to detention of drug to diseased colitis tissue.^100^ A supportive base for muco-adhesive nanodrug delivery system was suggested by Niebel et al who found that clodronate alone was ineffective in colitis therapy but proved efficient when it was combined with cationic nanoparticle polymethacrylate.^101^

#### Colon drug targeting using liposomes

The use of liposomes as carriers has improved drug delivery to colon. However, the liposomal formulations often target not only to diseased region but also affect healthy colon tissue.^102^ In order to achieve targeting of liposomal coated drug specifically to diseased tissue, they are combined with Abs (monoclonal antibodies) to produce immunoliposomes. Targeting colon using immunoliposomes proved highly efficient drug delivery than with liposomes deficit of target antibody ligand.^103^

One of the drawback of administering immuno-liposomes via oral route is that Abs are easily susceptible to destruction by acidic pH of the stomach and enzymes present.^104^ Also, the bilayer present in liposome gets digested by bile salts and enzymes if it has no additional protective covering.^105^ The penetrative power through mucosa helps liposome to be used effectively in treating inflammation of intestinal mucosal tissue in case of IBD.^106^ A study, to support the finding was done by Harel et al who experimentally proved that there induced colitis model compared to non-inflamed excised tissue.^107^

## Discussion

Colon targeted delivery system helps in providing both systemic and local effects. One of the main benefits of CDDS is that it has reduced systemic side effects. It also offers neutral pH, lowered drug dose, lowered enzymatic activity, Increased reactivity towards absorption enhancers, a longer traverse time, maintenance of drug in its unbroken form so close until it reaches the target site and drug supply only when required. Here, primary approaches are less specific when compared to novel approaches. Main element in the colon TDDS is the biodegradable polymers. Systems which make use of these polymers are more environment friendly for the peptide and protein drugs that cover down the unfavorable effects in the treatment of colonic diseases, amoebiasis and helminthiasis etc reducing the massive first pass effects of steroids and hence results in the detained absorption of drug used to treat angina, asthma and rheumatoid arthritis. The prevailing dissolution techniques are not appropriate for estimations. Therefore, researches are going on to crop up never dissolution techniques to estimate the colon specific drug delivery system.

## Future Perspective and Conclusion

The future perspective aim on the use of nanoscale materials and a combination of stimuli responsive polymers with biological systems which provide access to variations of new functions and properties. Hence new theories are required to advocate these developments and these new theories should be such that they should explain the newly discovered behavior in a smart fashion in order to meet the requirements of a particular application in specific. Another problem is to evolve a system that respond to multiple external stimuli in an ‘intellectual’ and expected manner. One of the promising systems for controlled drug delivery is smart polymers, due to its short half-life, increased susceptibility towards hepatic and gastric destruction, narrow ther apeutic window and increased medicinal activities at lower plasma concentrations. These drug delivery systems face many difficulties with regard to their development relating to drug safety, stability, kinetic associated with drug reliefs, circumstances under which drug system is transported in to the body and callosities towards the altering metabolic state. Efficiency of drug delivery system mainly through smart polymers for protein and peptide drugs has been experimented in many patients. These systems have also appeared as a possible approach for the control release of bioactive agents. Some of the most important futuristic applications of smart polymers are urine analysis using smart toilette and identification of various health issues. Due to the ability of smart polymers to detect the presence of certain biomarkers, it is being widely used for targeting a drug to cure specific disease conditions like in case of detection of anti-cancer agents to tumor cells, smart polymer sensitive to folate receptors are used hence smart polymers emerge as new preferment in the area of drug development due to its increasing need in controlment or site-specific delivering systems. However, a great fraction, still remains unexplored and hence there are tons of opportunities for research in order to identify an ideal drug delivery system. To conclude smart polymers, have tremendous capabilities both in biomedical as well as biotechnology applications if they overcome all these hurdles or barriers.

## Ethical Issues

Not applicable.

## Conflict of Interests

There is no conflict of interest.
